# 

**Published:** 1816-08

**Authors:** 


### CORRESPONDENCE.

The contemptible forgery of Dr. Baillie's name, for the purpose of imposing on the Editors and the Public, was no sooner seen than detected. A second attempt of the kind might, perhaps, meet with more severe treatment than a simple reprehension.

Mr. Foy's Case of Melancholia from a material Cause in the Brain; Mr. Bird's Case of diseased Brain; and Mr. Bailey's Case, with the dissection, shall appear in our next Number.

Reviews of Reid on Nervous Disorders; Waller on Incubus; and Whateley on the Cure of Wounds and Ulcers on the Legs, will also appear in the ensuing month.

Mr. Baillie on Hemeralopia is received

The promise of Communications from D. affords us much pleasure.—Mr. P's interesting Case, with the post mortem appearances; and Philo-Medicus on Pulmonary Hæmorrhage, shall be inserted in the fourth department of our Journal as soon as possible.

Mr. Howship's Observations on the Diseases of the Urinary Organs, Mr. Ring's Answer to Kinglake, and Dr. Scudamore's Work on Gout, have been received.

Authors or Publishers are requested to send a Copy of every new Work to the Editors (care of Mr. Thorne) as soon as possible after publication, that no time may be lost in laying an Analysis of it before the Public. The Editors conceive that such a mark of politeness on the part of Authors is well repaid by the *early* publicity which is consequently given to their Works.

